# Foundation first: a health-system readiness framework for responsible AI adoption in Somalia

**DOI:** 10.3389/fdgth.2026.1959721

**Published:** 2026-10-07

**Authors:** Hamze G. Dahir, Abdisalam Hassan Muse

**Affiliations:** 1 School of Public Health and Nutrition, College of Health Sciences, Amoud University, Borama, Somaliland, Somalia; 2 Research and Innovation Center, Amoud University, Borama, Somaliland, Somalia

**Keywords:** artificial intelligence, digital health, health equity, health systems strengthening, health technology implementation, implementation science, pastoralists, Somalia

## Abstract

Artificial intelligence (AI) is increasingly positioned as a mechanism for expanding access, strengthening surveillance, improving clinical decisions, and increasing health-system efficiency. Yet AI implementation is path-dependent: its value depends on the health-system, digital, institutional, and social foundations into which it is introduced. Somalia provides a critical test of this proposition because a dynamic telecommunications ecosystem coexists with uneven electricity, broadband use, primary care capacity, routine data quality, digital skills, and governance, particularly across rural, pastoralist, nomadic, displaced, and drought-affected populations. This Perspective develops a foundation-first AI implementation agenda for Somalia. It integrates the World Health Organization health-system building blocks, the Nonadoption, Abandonment, Scale-up, Spread and Sustainability framework, and an equity lens, while aligning proposed actions with the Sustainable Development Goals, Somalia's National Transformation Plan 2025–2029, Health Sector Strategic Plan III, and African Union Agenda 2063. The central argument is that AI should be treated as a capstone of digital transformation rather than its starting point. Reliable energy, inclusive connectivity, strong primary and community care, interoperable information systems, workforce capability, sustainable financing, and accountable governance should precede or accompany AI deployment. This sequencing can convert AI from a potentially exclusionary technology into a context-fit multiplier of health-system capacity.

## 1 Introduction

Artificial intelligence (AI) for health can be understood as the use of algorithmic systems that learn from data to perform or support tasks in clinical care, public health, administration, or research; digital health is the broader use of digital technologies, data, and connected systems to improve health and health services (1, 2). In this Perspective, health-system foundations are defined as the minimum service, workforce, information, technology, financing, leadership, and governance capacities required for a digital intervention to function reliably at scale. This definition builds on the World Health Organization (WHO) six health-system building blocks—service delivery, health workforce, health information, medical products and technologies, financing, and leadership/governance—and treats electricity and connectivity as cross-cutting implementation infrastructure rather than optional technical add-ons (3). Digital health implementation therefore refers not simply to installing a tool, but to integrating it into workflows, institutions, financing, regulation, and population needs so that it can be adopted, scaled, sustained, and evaluated in routine practice (4).

The policy lineage matters. The Millennium Development Goals (MDGs) concentrated global attention on child survival, maternal health, HIV/AIDS, malaria, and other communicable diseases, demonstrating the value of measurable targets but also exposing the limitations of disease-specific progress when underlying systems remain weak (5). The Sustainable Development Goals (SDGs) broadened this agenda: SDG 3.8 makes universal health coverage central, while SDGs 7, 9, 10, 13, and 17 connect health to reliable energy, infrastructure and innovation, reduced inequalities, climate resilience, and partnerships (6). AI may contribute to many SDG targets, but influential evidence also shows that it can worsen inequality or undermine sustainable development without appropriate governance and infrastructure (7). For Africa, Agenda 2063 similarly links healthy and well-nourished citizens with science, technology and innovation, quality basic services, and climate-resilient development (8). In low- and middle-income countries, responsible AI therefore requires explicit guardrails for user-centred design, local validation, equity, data quality, costs, and unintended system effects so that innovation helps rather than harms resource-limited populations (29). The Lancet and Financial Times Commission further argues that digital transformation should be governed as a public-purpose and rights-based process, with equity, inclusion, trust, and accountability embedded in national digital-health policy (30).

Somalia's current policy environment creates a timely opportunity to translate these global commitments into implementation choices. The National Transformation Plan (NTP) 2025–2029 is the country's primary development roadmap and organizes reform around transformational governance, sustainable economic transformation, social and human capital transformation, and environment and climate resilience (9). The Health Sector Strategic Plan III (HSSP III) 2022–2026 emphasizes essential services, primary health care and progress toward universal health coverage (10). These priorities intersect directly with digital health: resilient public infrastructure, stronger institutions, human capital, and integrated service delivery are prerequisites for safe AI rather than parallel policy agendas.

Globally, digital health has moved from isolated eHealth projects toward national architecture, interoperability, and responsible AI. WHO extended its Global Strategy on Digital Health through 2027 and reported that 129 countries had established national digital health strategies, while governance of AI and cross-border interoperability have become explicit priorities (1, 11). Across Africa, the Africa Centres for Disease Control and Prevention (Africa CDC) Digital Transformation Strategy seeks to strengthen public health systems through digitalization, but evidence continues to identify fragmented, partner-driven applications, interoperability gaps, limited infrastructure, and workforce constraints as barriers to system-wide impact (12–14). In the Horn and East African region, Ethiopia illustrates both the potential and the implementation challenge: a systematic review documented expanding use of mHealth, electronic records, telemedicine and AI, while evaluation of mobile health services in the Somali and Afar regions showed that services designed around pastoralist mobility improved reach but remained dependent on appropriate implementation guidance and system support (15, 16).

At the Somali level, recent scholarship has reviewed telemedicine, mobile health, electronic records and AI and concluded that digital health could improve access if accompanied by policy, infrastructure, coordination, workforce development and evaluation (17). A 2025 multi-stakeholder analysis of the NTP health agenda likewise identified governance, workforce, financing, service delivery and system integration as priorities for post-conflict health transformation (18). The unresolved question is therefore not whether digital health is promising, but how AI should be sequenced within Somalia's fragile, unequal, mobile and climate-exposed health system. The specific contribution of this Perspective is to convert that gap into an implementation framework. Its purpose is to define the foundations required for equitable AI-enabled care, align them with national and continental policy, and propose practical criteria for deciding when AI adds system value rather than technological complexity.

## 2 Theoretical and analytical framework

This Perspective uses a Foundation-First AI Implementation Framework that synthesizes three complementary lenses. Rather than treating these frameworks as parallel or merely additive, we position them as a nested hierarchy. First, the WHO health-system building blocks identify the structural prerequisites (the ‘what’) that AI inherits: service delivery, workforce, information, medicines and technologies, financing, and governance (3). Second, Greenhalgh et al.'s Nonadoption, Abandonment, Scale-up, Spread and Sustainability (NASSS) framework treats technology implementation as a dynamic sociotechnical process (the ‘how’) involving the technology, its value proposition, users, organizations, the wider institutional context, and adaptation over time (4). Third, an equity and climate lens provides the boundary conditions (the ‘who’ and ‘where’), asking who can actually use and benefit from a digital intervention when electricity, connectivity, device access, literacy, disability, geography, displacement, gender, income, and mobility differ across populations (19).

The framework distinguishes established components from the authors’ synthesis. The WHO health-system building blocks, the NASSS domains, and the equity and climate-resilience considerations are adapted from the cited literature; they are not presented as new theories. The original contribution is the Foundation-First AI Implementation Framework: a Somalia-focused readiness sequence that links those lenses through four decision criteria—readiness, implementation fit, equity reach, and sustainability—and applies them to policy-aligned AI choices. The proposed sequence and implementation tests are therefore analytical recommendations for this Perspective, not a validated prediction model.

The analytical logic is sequential. Foundational readiness asks whether the service, energy, connectivity, data, workforce and governance conditions needed for a proposed AI use case exist. Implementation fit asks whether the technology can be integrated into workflows and organizations under real operating conditions. Equity reach asks whether benefits extend beyond already connected urban users to rural, pastoralist, displaced and otherwise underserved populations. Sustainability asks whether national institutions can finance, maintain, regulate, monitor, modify and, when necessary, discontinue the technology after pilot funding ends. These four criteria—readiness, fit, equity and sustainability—organize the argument and recommendations below. Table 1 maps the analytical framework to relevant policy commitments.

**Table 1:** Analytical framework and policy alignment for foundation-first AI implementation in Somalia.

Analytical lens	Core question	Policy alignment	Somali implication
WHO health-system building blocks	Are service delivery, workforce, information, technology, financing and governance strong enough to support the intervention?	SDG 3; HSSP III; NTP social and human capital transformation; Agenda 2063 Goal 3	Treat primary care, routine information, workforce and governance as prerequisites for AI.
NASSS implementation framework	Does the technology offer a clear value proposition, and can it be adopted by users, integrated into organizational workflows, supported by the wider institutional context, and adapted over time?	NTP transformational governance; SDG 9 and 17	Evaluate workflow fit, value, organizational capacity, procurement, lifecycle financing and adaptation.
Equity lens	Who gains access and who remains excluded because of geography, mobility, disability, income, literacy, devices, power or connectivity?	SDGs 7 and 10; Agenda 2063 inclusive growth and basic services	Design for rural, pastoralist, nomadic, displaced and drought-affected populations, not only connected urban users.
Climate-resilience lens	Will the intervention continue to function during drought, displacement, infrastructure failure or interrupted connectivity?	SDG 13; NTP environment and climate resilience; Agenda 2063 Goal 7	Require offline capability, delayed synchronization, portable records and resilient energy/connectivity.

We acknowledge an inherent tension between the non-linear, complex systems approach of the original NASSS framework and the linear sequence implied by a ‘foundation-first’ model. This framework resolves that tension by proposing a two-phase reality: the establishment of structural prerequisites (e.g., electricity, basic data architecture, clinical referral pathways) must logically and sequentially precede advanced AI deployment to prevent immediate failure. However, once those baseline foundations are met, the subsequent AI implementation process—adoption, workflow integration, scaling, and adaptation—becomes a highly iterative, non-linear, and complex process exactly as NASSS describes.

## 3 Historical and current status: from global digital health to the Somali context

The historical trajectory of digital health has shifted from stand-alone telemedicine and disease-specific information systems toward interoperable national platforms and, more recently, AI-enabled decision support. That shift is consequential for low-resource settings because the value of advanced analytics depends on the maturity of the layers beneath them. WHO's current strategy explicitly states that national digital health initiatives require integrated financial, organizational, human and technological resources (1). The World Bank's 2025 AI foundations framework similarly emphasizes connectivity, compute, context and competency as conditions for inclusive AI adoption (20). A 2025 implementation review further argues that clinical AI must be treated as a lifecycle process involving co-design, deployment, monitoring, governance and maintenance rather than model performance alone (21). This pattern is not unique to sub-Saharan Africa: qualitative evidence from health-AI stakeholders across seven Southeast Asian countries similarly identified investment in digital infrastructure, technology governance, and data governance as foundational conditions for successful AI adoption (31).

Continental evidence points in the same direction. Africa CDC positions digital transformation as a mechanism for stronger public health systems, yet systematic review evidence from African health information exchange shows that policy commitments to interoperability often outpace real deployment and adoption (12, 14). Health-system strengthening literature in Africa has also criticized vertical, partner-specific digital investments that create isolated applications instead of reusable national infrastructure (13). The implication is that Africa's challenge is no longer simply to acquire digital tools; it is to develop country-owned architectures capable of integrating them. More recently, an Africa CDC Primary Health Care Digitalization Expert Committee outcome statement reported that only about 30% of African health systems were digitalized and that overseas development assistance had declined by nearly 70% since 2021; because these are continental advocacy estimates rather than Somalia-specific readiness measures, they reinforce the urgency of domestically financed digital foundations but should not be interpreted as direct estimates for Somalia (35).

The Horn of Africa adds a distinctive geographic and livelihood dimension. Evidence from Ethiopia shows that digital technologies can reach large populations, but implementation is uneven across technology types and settings (15). In pastoralist areas of the Somali and Afar regions, mobile health services were more effective when delivery followed population movement and local settlement patterns rather than assuming fixed catchment populations (16). This regional evidence is directly relevant to Somalia, where health-system design must accommodate mobility instead of treating it as an exceptional condition.

Somalia's local digital landscape is therefore paradoxical. Telecommunications and mobile money demonstrate substantial private-sector innovation, yet active broadband use, electricity access, health-facility readiness and routine information capacity remain uneven. International Telecommunication Union estimates for 2024 reported active mobile-broadband subscriptions of about 13 per 100 people, 3G population coverage of 83%, LTE/WiMAX coverage of 61%, mobile-phone ownership of 47.9%, and internet use of 27.9% (22). These national, single-year indicators describe different dimensions of connectivity: population coverage does not establish reliable service, affordability, device access, actual use, or readiness at individual health facilities, and national averages may conceal substantial geographic and socioeconomic variation. World Bank data similarly reported national electricity access of 50.3% in 2023, but this aggregate does not capture reliability, affordability, facility-level availability, or rural variation (23). The figures therefore indicate plausible infrastructural constraints rather than deterministic causal effects. In combination, limited use and uncertain service reliability create a substantial and foreseeable risk that cloud-dependent AI could experience localized interruptions or widen effective-access gaps unless deployments include facility-level readiness assessment, resilient power, low-bandwidth operation, and offline functionality.

Available Somalia-specific evidence also points to the foundations that require attention before advanced AI can be assessed. The World Bank reports very low national access to electricity, while ITU indicators show a substantial gap between population coverage and actual mobile-broadband use (22, 23). National policy evidence identifies fragmented information systems, workforce constraints, financing limitations, and weak coordination as continuing health-sector barriers (17, 18). The Somalia Data Protection Act provides a legal basis for data governance, but the existence of legislation should not be interpreted as evidence that facility-level privacy, cybersecurity, interoperability, or enforcement capacity is already adequate (36). These sources do not provide a nationally representative readiness score; they establish a policy and infrastructure context that the proposed framework converts into testable implementation requirements.

Fragility and climate shocks add further constraints. WHO's 2026 public health situation analysis describes conflict, displacement, weak infrastructure, recurrent climate shocks and constrained access to services as interacting determinants of health need (24). A Ministry of Health-WHO-UNICEF mortality analysis estimated approximately 71,100 drought-related excess deaths between January 2022 and June 2024, with 41% among children younger than five years (25). Such shocks change where people live, interrupt referral and follow-up, overload receiving facilities and destabilize the assumptions of fixed-location digital records. In Somalia, resilience to mobility and infrastructure interruption is therefore a core technical requirement, not a secondary feature.

## 4 Somalia's implementation paradox: AI amplifies the system it inherits

AI cannot independently create medicines, referral transport, trained clinicians, reliable laboratories, electricity, complete records or public trust. It operates on the infrastructure, workflows, data and institutions already available. An outbreak-prediction model cannot compensate for districts that fail to report cases; a maternal decision-support tool cannot prevent an avoidable death when emergency referral is unavailable; and a cloud-based teleconsultation platform cannot serve a community when power and connectivity repeatedly fail. The relevant unit of analysis is therefore the full implementation chain rather than the algorithm alone.

Somalia already has assets that should be strengthened rather than bypassed. WHO reports that the Integrated Disease Surveillance and Response system uses DHIS2 to support national surveillance reporting and outbreak detection (26). The new Community Health Strategy 2025–2029 places a harmonized and professionalized community health workforce, including Female Health Workers, at the center of household-level primary care (27). These systems provide a practical platform for context-fit AI: decision support can be embedded in existing community and surveillance workflows instead of creating parallel donor-dependent applications. A 2026 Somalia-focused commentary on mental-health services reaches a compatible conclusion, positioning AI as a system-support tool for task-shared care, supervision, referral, and routine DHIS2 data use rather than as a substitute for clinicians or core services (32).

The equity risk is equally important. AI-enabled services can expand access, but the innovation curve can reproduce existing social gradients when devices, connectivity, literacy and service availability are unequally distributed (19). In Somalia, a connected urban patient may benefit rapidly from AI-assisted services while a pastoralist family, remote household or newly displaced person remains nominally covered by a national strategy but practically excluded. Effective access—not application counts, registrations or pilot numbers—should therefore be the primary equity outcome.

## 5 A foundation-first implementation agenda for Somalia

The first priority is reliable service infrastructure. Primary and community care, referral pathways, essential medicines, diagnostics and emergency transport must remain the clinical substrate of any AI intervention. Facility electrification—including solar-plus-storage where appropriate—should be treated as health-system and digital-health infrastructure because it simultaneously supports cold chains, diagnostics, lighting, communications and computing. Connectivity planning should likewise prioritize primary-care facilities, rural settlements, pastoralist corridors and displacement sites and should require low-bandwidth and offline-capable design even as network coverage improves.

The second priority is national information architecture. AI requires consistent, interpretable and governed data. Somalia should therefore strengthen DHIS2 and other routine systems, interoperability standards, data-quality assurance, metadata, aligned identifiers where appropriate, and clear rules for secondary data use before scaling advanced analytics. African experience shows that health information exchange and reusable digital platforms are essential to avoid fragmented vertical systems (13, 14).

The third priority is human and institutional capability. Frontline health workers, managers, policymakers and technical teams need digital and AI literacy appropriate to their roles. AI should augment workers through approved guidelines, standardized triage, nutrition screening, immunization follow-up, and anomaly detection, supply forecasting and evidence retrieval rather than displace clinical accountability. Every intervention should also include lifecycle financing, maintenance, cybersecurity, procurement standards, vendor accountability, auditability and local technical capacity. Table 2 translates these principles into operational priorities.

**Table 2:** Foundation-first sequence for equitable AI-enabled health technology implementation in Somalia.

Foundation	Implementation bottleneck	Priority action	AI implementation test
Essential health services	Uneven primary care, referral, diagnostics and medicines	Strengthen PHC, community care, referral and essential commodities	Does AI improve an existing care pathway rather than substitute for missing care?
Electricity and connectivity	Intermittent power; rural and last-mile connectivity gaps	Prioritize resilient power, solar storage, health-facility connectivity and offline design	Can the tool function during power/network interruptions and synchronize safely later?
Health information systems	Fragmented applications and variable data completeness	Strengthen DHIS2/routine systems, interoperability, standards and data quality	Are input data sufficiently complete, structured, representative and governed?
Workforce and organizations	Digital skills gaps and workflow disruption	Build role-specific AI literacy, co-design and workflow integration	Do users understand outputs, limits, escalation pathways and human oversight?
Governance and financing	Privacy, cybersecurity, procurement and donor dependence	Establish national rules, validation, incident reporting, lifecycle financing and exit plans	Can Somali institutions regulate, finance, maintain, audit and discontinue the tool?
Equity and resilience	Risk of urban bias and exclusion of mobile populations	Monitor access/outcomes by geography and vulnerability; design for mobility and crisis	Does implementation reduce rather than widen effective-access gaps?

## 6 What fit-for-context AI should look like

The appropriate AI model for Somalia should be defined less by computational novelty than by reliability under constraint. Applications should minimize data and bandwidth requirements, continue essential functions during connectivity interruptions, synchronize when networks return, operate on affordable devices, and use local processing where feasible. Interfaces should accommodate Somali-language use, varying literacy and the realities of community and mobile health work. These design choices are not concessions to a temporary infrastructure deficit; they are requirements for equitable implementation in a country where mobility and climate shocks will continue to shape service delivery.

Use cases should be selected by health-system need. Potential early applications include identifying incomplete surveillance reports, supporting community triage against approved protocols, flagging missed immunizations, assisting nutrition screening, forecasting essential commodities, detecting anomalies in routine data and helping clinicians retrieve guidelines. Each proposal should answer four questions before procurement: What specific bottleneck does it solve? Why is AI preferable to a simpler digital or non-digital intervention? What evidence will demonstrate benefit and absence of harm in the Somali context? Can the intervention be governed, maintained and financed nationally after external support ends? Recent implementation science for healthcare AI reinforces the importance of co-design, real-world clinical utility, governance and lifecycle monitoring rather than accuracy metrics alone (21). A concrete local example is Benadir's electronic immunization registry, which has been reported to improve vaccination tracking and reduce dropout; this should be understood as an existing digital foundation on which carefully evaluated decision support might later be layered, not as evidence that AI itself produced those gains (34).

To illustrate this framework in practice, consider the hypothetical deployment of an AI-assisted smartphone application for malnutrition screening (e.g., using computer vision for anthropometric measurements) among pastoralist communities. Applying the foundation-first framework:

1) WHO Building Blocks dictate that before deploying the AI, there must be a reliable supply of therapeutic food, trained community health workers (CHWs), and solar-powered charging stations;
2) NASSS requires that the tool fits the CHWs’ daily workflow and that the Ministry of Health has the organizational capacity to finance the software licenses post-pilot;
3) The Equity and Climate lens mandates that the app must function entirely offline during nomadic movements and synchronize data only when the CHW returns to a network coverage area. Without these sequenced foundations, the AI tool would merely identify malnourished children who cannot be treated, or fail entirely when internet access drops. Under Table 2's AI implementation test, this use case would therefore receive a conditional ‘not ready for scale’ conclusion until the treatment-and-referral pathway, resilient power and offline synchronization, representative and governed input data, trained-user oversight, lifecycle financing, and equity monitoring are demonstrated in routine conditions.

## 7 Governance, ethics, and accountability must precede scale

AI introduces risks that infrastructure alone cannot resolve. Health data are sensitive; models may encode bias; proprietary systems may be opaque; and models trained elsewhere may perform poorly in Somali populations. WHO guidance places autonomy, safety, transparency, accountability, inclusiveness and sustainability at the center of AI governance (2). Guidance for large multimodal models further emphasizes stakeholder participation, well-defined tasks, regulation, impact assessment and post-deployment monitoring (28). Somalia-specific scholarship also identifies AI-generated and algorithmically amplified health misinformation as an emerging threat to public trust, health behaviour, and epidemic preparedness, particularly where Somali-language verification resources and digital-health literacy are limited (33).

Somalia therefore needs national rules for data stewardship, privacy, cybersecurity, interoperability, procurement, validation, human oversight, incident reporting, algorithmic change management and accountability. These rules are especially important in a plural health sector where government, humanitarian agencies, nongovernmental organizations, donors and private providers may procure technologies independently. The implementation test should extend beyond whether an AI tool works in a pilot: decision-makers should ask whether national institutions can understand it, regulate it, finance it, maintain it, audit it, adapt it and stop using it safely when necessary. This recommendation should not be read as implying a complete legal vacuum: Somalia's Data Protection Act No. 005 of 2023 established general requirements for protecting and processing personal data and created the Somalia Data Protection Authority (36). However, general data-protection law does not by itself supply a complete health-AI governance regime; AI-specific provisions remain needed for clinical validation, algorithmic transparency, bias assessment, human review, procurement, change control, liability, post-deployment surveillance, and management of AI-generated misinformation (32, 33, 36).

## 8 Discussion: policy implications and novelty

The central implication is that AI readiness should be assessed as a health-system property rather than as a technology inventory. Somalia has plausible use cases for surveillance, remote support, decision assistance and resource allocation, but the same geographic, institutional and climate-related constraints increase implementation risk. Accordingly, funders and implementers should judge each proposal by whether it strengthens a defined care or public-health pathway, functions under real operating conditions, and includes an accountable plan for equity, maintenance and evaluation.

The novelty of this Perspective lies in its sequencing argument and analytical synthesis. Existing Somalia-focused literature has established the promise and challenges of digital health technologies (17), and recent national policy research has identified broad health-system priorities under the NTP (18). This paper connects those streams to implementation science by combining WHO health-system foundations, NASSS, equity, climate resilience and explicit alignment with the SDGs, NTP 2025–2029 and Agenda 2063. The resulting framework shifts the policy question from ‘Which AI tool should Somalia adopt?’ to ‘Which foundations and implementation conditions must be present for a defined AI use case to improve equitable health outcomes?’ References 17–18 alone establish neither a use-case-level readiness sequence nor a decision rule for withholding, adapting, or scaling a proposed AI intervention. By nesting structural readiness within NASSS implementation dynamics and applying equity and climate conditions, this paper derives the operational recommendation that an AI use case should not proceed to scale until its specific care pathway, infrastructure, data, workforce, governance, financing, and resilience tests are satisfied; when they are not, investment should first target the failed foundation.

This framing also changes how donors and implementing partners should evaluate innovation. Reliable electricity, interoperable data, workforce capability and community-health workflows are no-regret investments because they improve care with or without AI. A modest offline decision-support function embedded in a national workflow may produce more durable value than a sophisticated platform requiring continuous cloud access, proprietary data structures and temporary external expertise. Implementation success should therefore be judged by system fit, equity, sustainability, governance and measurable improvement in care—not technological sophistication.

While this framework is designed specifically for Somalia, its boundary conditions make it generalizable to other Fragile, Conflict-affected, and Vulnerable (FCV) settings, particularly across the Sahel and the Horn of Africa, where high pastoralist populations and climate shocks intersect with weak health infrastructure. Conversely, this foundation-first sequencing may be less applicable to middle- or high-income settings with stable power grids, mature electronic health records, and established regulatory bodies, where implementation can focus more immediately on algorithmic performance and advanced clinical integration.

## 9 Conclusion

Somalia does not need to choose between strengthening its health system and participating in the AI era; it needs to establish the correct order. Digital transformation should begin with reliable primary and community care, resilient electricity and connectivity, interoperable information systems, capable health workers, sustainable financing and trusted governance. These foundations are consistent with the SDGs, Somalia's NTP and health-sector strategies, and Agenda 2063, and they are especially important for rural, pastoralist, nomadic, displaced and drought-affected populations.

AI should then be introduced where it addresses a defined system bottleneck, functions under real operating conditions, has a credible evidence and monitoring plan, and can be governed and maintained nationally. The defining measure of Somalia's digital transformation should not be the number of AI pilots deployed. It should be whether technology helps a progressively stronger health system reach people more equitably, reliably and sustainably. AI cannot substitute for health-system foundations; when placed on top of them, however, it can become a meaningful multiplier of national health-system capacity.

## Data Availability

The original contributions presented in the study are included in the article/Supplementary Material, further inquiries can be directed to the corresponding author.
